# IgD ^+^ age‐associated B cells are the progenitors of the main T‐independent B cell response to infection that generates protective Ab and can be induced by an inactivated vaccine in the aged

**DOI:** 10.1111/acel.13705

**Published:** 2022-09-02

**Authors:** Olivia Kugler‐Umana, Wenliang Zhang, Yi Kuang, Jialing Liang, Catherine H. Castonguay, Susan L. Tonkonogy, Ann Marshak‐Rothstein, Priyadharshini Devarajan, Susan L. Swain

**Affiliations:** ^1^ Department of Pathology University of Massachusetts Chan Medical School Worcester Massachusetts USA; ^2^ College of Veterinary Medicine North Carolina State University Raleigh North Carolina USA; ^3^ Department of Medicine University of Massachusetts Chan Medical School Worcester Massachusetts USA

**Keywords:** adaptive immunity, age‐associated B cells, aging, influenza, TLR signals

## Abstract

Age‐associated B cells (ABC) accumulate with age and are associated with autoimmunity and chronic infection. However, their contributions to acute infection in the aged and their developmental pathways are unclear. We find that the response against influenza A virus infection in aged mice is dominated by a Fas^+^GL7^−^ effector B cell population we call infection‐induced ABC (iABC). Most iABC express IgM and include antibody‐secreting cells in the spleen, lung, and bone marrow. We find that in response to influenza, IgD^+^CD21^−^CD23^−^ABC are the precursors of iABC and become memory B cells. These IgD^+^ABC develop in germ‐free mice, so are independent of foreign antigen recognition. The response of ABC to influenza infection, resulting in iABC, is T cell independent and requires both extrinsic TLR7 and TLR9 signals. In response to influenza infection, IgD^+^ABC can induce a faster recovery of weight and higher total anti‐influenza IgG and IgM titers that can neutralize virus. Immunization with whole inactivated virus also generates iABC in aged mice. Thus, in unimmunized aged mice, whose other B and T cell responses have waned, IgD^+^ABC are likely the naive B cells with the potential to become Ab‐secreting cells and to provide protection from infection in the aged.

## Graphical abstract

A key challenge for the aged is overcoming respiratory infections caused by fast‐mutating and novel viruses (influenza, SARS‐CoV2) because of their impaired ability to generate effective new immune responses. New vaccines are also less effective in the aged. We show that the naive IgD^+^ABC (Age‐associated B cells), a population specialized to mount responses in an aging immune environment, become a population of effectors that differentiate into antibody‐secreting cells and memory B cells against influenza infection that likely contribute to protection.

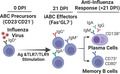

## 1 INTRODUCTION

In elderly patients, lower respiratory tract infections caused by viruses such as influenza and SARS‐CoV‐2, are the most common cause of hospitalizations (Bartleson et al., 2021; Kline & Bowdish, 2016). In the US, over half of influenza‐related hospitalizations and deaths and over 80% of COVID‐related deaths, were in patients over the age of 65 (Bartleson et al., 2021; Kline & Bowdish, 2016). Immune response to infections in aged populations are often weak and most current influenza vaccines fail to induce sufficient protective immunity to prevent severe illness (Frasca & Blomberg, 2020; McElhaney et al., 2016). Defining the changes in the immune system with age and in response to influenza infection is likely to provide insights into the heightened susceptibility of elderly patients to new infection with rapidly mutating viruses like influenza and other ssRNA viruses. Determining the mechanisms that promote the most effective immune responses in the aged, should inform strategies to improve vaccines and therapies for the aged.

The immune system undergoes many changes with age that contribute to reduced responses to infections and to immunizations (Frasca & Blomberg, 2020; Shaw et al., 2013; Swain et al., 2021). Responses mediated by innate cells are reduced (Shaw et al., 2013; Wong et al., 2017) with age. Multiple steps in the development of adaptive responses of T and B cells are also impaired (Frasca & Blomberg, 2020; Haynes & Swain, 2006). Aged mice have a much smaller pool of naive CD4 T cells, and thus, have a more restricted TCR repertoire (Dorshkind & Swain, 2009). Aged naive CD4 cells respond less vigorously to antigen and develop fewer CD4 T cell effectors (Chinn et al., 2012; Jones et al., 2010; Shifrut et al., 2013; Zhang et al., 2014), including T follicular helper (T_FH_) cells (Eaton et al., 2004; McElhaney, 2011). The aged effector T cells develop little T cell memory (Zhou & McElhaney, 2011). Like T cells, the generation of immature B cells in the bone marrow (BM) decreases with age (Scholz et al., 2013), so the naive mature B cell pool consists of longer‐lived, older follicular B cells (FOB) and marginal zone B cells (MZB; Guerrettaz et al., 2008; Gibson et al., 2009) that respond less vigorously to antigenic challenge than B cells from younger individuals (Frasca & Blomberg, 2020). In responses to new or mutated pathogens, these impairments in conventional naive B cell and T_FH_ responses cause decreased germinal center B cell (GCB) responses (Brahmakshatriya et al., 2017; Haynes et al., 2004; Jones et al., 2010) with fewer isotype‐switched Ab‐secreting cells (AbSC), fewer long‐lived PC (LLPC) and fewer memory B cells (B_mem_) (Brahmakshatriya et al., 2017; Eaton et al., 2008; Lefebvre et al., 2016; Zhang et al., 2014). This leads to less effective clearance of virus and less durable protective memory (Frasca & Blomberg, 2020). Since responses of naive B cells to novel antigens presented by new variants of viruses and vaccines are poor in the aged (Frasca & Blomberg, 2020; Scholz et al., 2013), there is an increased reliance on previously generated memory B cell responses (Devarajan & Swain, 2019). Although there are fewer naive FOB cells with age, the total number of B cells is similar in young and aged mice (Scholz et al., 2013), raising the possibility that additional subsets of B cells might exist in aged animals.

Michael Cancro's group identified a novel subset of splenic B cells that accumulated with age in unimmunized mice (Hao et al., 2011) and were defined by lack of expression of CD21 and CD23 (Hao et al., 2011; Ratliff et al., 2013). B cells of that phenotype were subsequently identified in humans (Rubtsov et al., 2011) and were also called age‐associated B cells (ABC). Experiments using ABC from unimmunized mice, indicated that addition of TLR7 or TLR9 stimulation paired with anti‐μ activation was needed to induce their proliferation in vitro (Hao et al., 2011). Pippa Marrack and colleagues simultaneously described a murine CD21^−^CD11c^+^CD11b^+^ population which increased with age and also was called ABC (Rubtsov et al., 2011). They found these CD11c^+^ABC accumulated in unimmunized aged and young autoimmune‐prone mice (Manni et al., 2018; Rubtsov et al., 2011, 2013) and that they played a key role in autoimmunity. The Marrack ABC resembled extrafollicular (EF) B cells, or atypical B cells, that develop in autoimmunity (Jenks et al., 2018; Rubtsova et al., 2017) and in chronic infections in humans (Courey‐Ghaouzi et al., 2022; Sutton et al., 2021) as all share CD11c and T‐bet expression. Thus, there is compelling evidence that ABC are a unique B cell population that develops with age and that ABC play key roles in autoimmunity and are associated with aged responses to chronic infection in both mice and humans. In contrast to ABC in unimmunized aged mice, which we show here are likely naive B cells, ABC characterized in chronically infected/autoimmune human patients are likely effector or memory B cells. Therefore, cells called ABC probably encompass B cells at multiple stages of differentiation (Kugler‐Umana et al., 2020).

Cancro has suggested that CD21^−^CD23^−^ABC are an antigen (Ag)‐experienced memory B cell population (Cancro, 2020; Russell Knode et al., 2017), since they require signals from CD40L, and thus, likely cognate interaction with CD4 helper T cells for their development (Russell Knode et al., 2017) and collectively contain more somatic mutations in their B cell receptors than naive follicular B cells (Russell Knode et al., 2017). However, we note that in unimmunized aged mice, the majority of ABC still express surface IgD (Swain et al., 2017) and express a diverse B cell receptor repertoire (Russell Knode et al., 2017), a phenotype more compatible with a naive population of B cells.

The extent of the role of ABC in acute infections and their contribution to anti‐pathogen immunity are not well‐defined. Given that the ABC population becomes a larger B cell subset as mice get older, making up more than one‐third of B cells in the aged mice (18–20 M) (Hao et al., 2011), and that FOB at this age respond very poorly (Frasca & Blomberg, 2020; Scholz et al., 2013), we postulated that ABC might make an important contribution to protective immunity and provide an alternate pathway to Ab production to pathogens. Thus, we set out to more clearly define the subset(s) of ABC in unimmunized aged mice that responds to infection, the mechanisms that drive their response and whether they give rise to protective AbSC that combat infection.

We analyzed the kinetics of B cell responses following influenza A virus (IAV) infection of aged versus young mice (Brahmakshatriya et al., 2017; Lefebvre et al., 2016) and found that aged mice developed an effector B cell population that expressed Fas but not GL7 (iABC). This population was the dominant B cell response in aged mice and it peaked at 21 days post‐infection (dpi) in the spleen, BM, and lung. Many of the iABC became AbSC (CD138^+^) that expressed IgM and published ABC markers, with small subsets expressing IgG2b and IgA. The CD21^−^CD23^−^ABC in aged naive wild‐type mice included both surface IgD^+^ and IgD^−^ B cells, and we found the IgD^+^ABC are the precursors of the great majority of iABC generated by influenza infection. The generation of iABC from IgD^+^ABC did not require T cell help but required both extrinsic TLR7 and TLR9 signals. Unlike iABC, the development of precursor ABC in aged mice did not require endosomal TLR and occurred in germ‐free (GF) mice in the absence of foreign Ag. This suggests that ABC development is part of an intrinsic program of aging and that IgD^+^ABC are most likely naive cells. We found that IgD^+^ABC‐derived AbSC can make influenza‐specific IgM and IgG Ab, which can neutralize influenza and can accelerate recovery from infection‐induced weight loss. Importantly, immunization with whole inactivated PR8 influenza virus (WIV), generated an equivalent iABC response in aged mice. Thus, we speculate that in response to infection, the IgD^+^ABC population in aged individuals differentiates into AbSC that provide Ab‐mediated protection and that in the aged ABC provide an alternate B cell pathway to protect against novel influenzas, and likely against SARS‐CoV‐2 and other viruses that depend on naive B cell responses.

## 2 RESULTS

### 2.1 In aged mice, iABC are the dominant responding B cells to IAV

**FIGURE 1:**
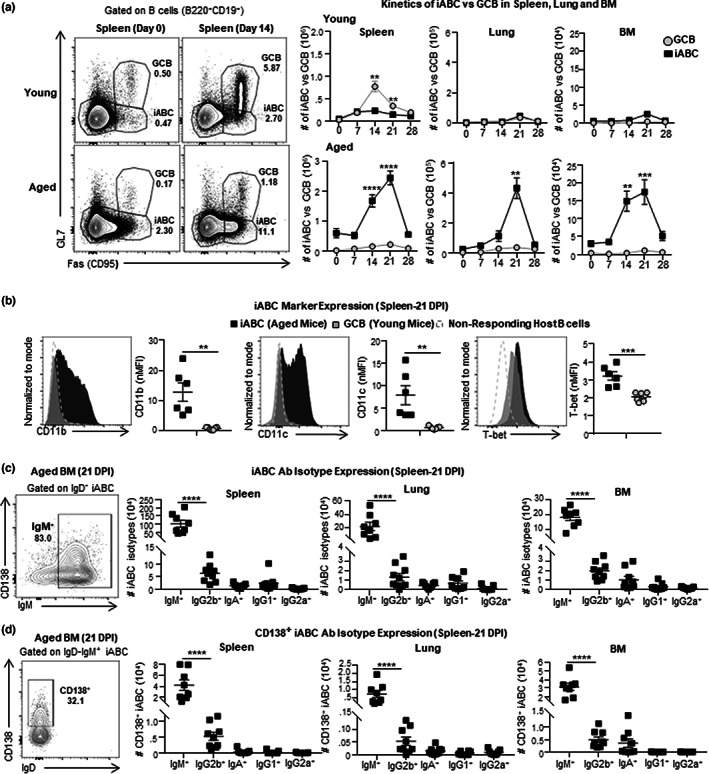
Kinetics of aged B cell effectors and Ab response. (a) Experimental Design: Aged (18–20 M) and young (3–4 M) B6 mice, were infected i.n. with 0.3 LD_50_ (25 PFU) PR8 (IAV) and sacrificed at 0, 7, 14, 21, and 28 dpi for analysis of B cell responses in the spleen, lungs and bone marrow (BM) by flow cytometry. Representative FACS plots indicate responding B cells by Fas and GL7 expression cells (left) and the kinetics of iABC(Fas^+^GL7^−^) versus GCB(Fas^+^GL7^+^) response is expressed as total cell numbers per organ in the spleen, lung and BM of young and aged mice. (b) Representative histograms and normalized MFI showing CD11c, CD11b, and T‐bet expression in iABC in aged mice (black) versus GCB in young mice (grey) response in the spleen. (c) Representative FACS plots and total numbers of IgM^+^, IgG2b^+^, IgA^+^, IgG1^+^, and IgG2a^+^iABC in spleen, lung and BM at 21 dpi. (d) Representative FACS plots and total numbers of IgM^+^CD138^+^, IgG2b^+^CD138^+^, IgA^+^CD138^+^, IgG1^+^CD138^+^, and IgG2a^+^CD138^+^iABC in spleen, lung and BM at 21 dpi (*n*=6–12 pooled from 2 to 3 separate experiments) Error bars represent SEM. Statistical significance determined by two‐tailed, unpaired Student's *t*‐test; *p < 0.05; **p < 0.01; ***p < 0.001, ****p < 0.0001

To better understand the features that distinguish B cell response in young (3–4 M) and aged (18–20 M) C57BL/6 mice, we monitored the in vivo kinetics of their B cell response to IAV infection. The young mice developed a robust GCB population, defined by expression of both Fas and GL7, that peaked at 14 dpi and was found in the spleen, but not lung or bone marrow (BM) (Figures 1a and S1a; Lefebvre et al., 2016). In contrast, in aged mice, we found very few GCB (Figures 1a and S1a) and instead they developed a dominant Fas^+^GL7^−^ population, which we termed infection‐induced age‐associated B cells (iABC). The iABC peaked at 21 dpi and were found in spleen, lung, and BM (Figures 1a and S1a). As expected, neither iABC nor GCB developed in the absence of IAV infection (0 dpi; Figure 1a). These results demonstrate that in naive aged mice infected with IAV, generation of iABC is the dominant outcome of the B cell response and hence the most likely source of protective Ab. Previous studies found ABC effector B cells linked to autoimmunity and chronic infections express CD11c, T‐bet, and CD11b (Jenks et al., 2018; Kugler‐Umana et al., 2020; Phalke & Marrack, 2018) which became the accepted markers of ABC. Here, we found in aged mice, that the iABC responding to influenza infection in the spleen, also expressed CD11c, T‐bet, and CD11b. Additionally, iABC in aged mice expressed these markers at higher levels compared with GCB in young mice at 21 dpi (Figure 1b). This suggests that many iABC share these signature phenotypes with those previously identified ABC effectors (Kugler‐Umana et al., 2020).

### 2.2 iABC include CD138 ^+^ plasma cells, that express mostly IgM with smaller IgG2b ^+^ and IgA ^+^ subsets

We asked if iABC include isotype‐switched B cells that express an AbSC phenotype (CD138^+^). At 21 dpi, we found that over 80% of responding iABC (IgD^−^Fas^+^GL7^−^) in the spleen, lung, and BM, expressed intracellular IgM (Figure 1c). About one‐third of IgM^+^iABC co‐expressed CD138^+^ in the BM, indicating that IgM^+^iABC include AbSC (Figure 1d). Therefore, the largest fraction of the iABC generated by IAV infection are IgD^−^IgM^+^ responding B cells which have not switched to other isotypes. This is consistent with the fact that influenza‐specific IgG antibodies decrease with age (Lefebvre et al., 2016). Further analysis of the iABC revealed a population of IgG2b^+^iABC in the lung (11–15%) and spleen (2–7%), and smaller populations of iABC expressing IgG1(1–4%) and IgA (2–6%) in the lungs (Figures 1c and S1b). The presence of AbSC among iABC in the lung is important since this is where influenza replicates. We did not detect any intracellular IgG2c (Figures 1c and S1b). Amongst IgD^−^IgM^−^iABC in the BM of aged mice, we detected IgG2b^+^iABC, as well as smaller populations of IgG1^+^ and IgA^+^ iABC (Figure S1b). Thus, the iABC response to IAV infection can develop AbSC of several isotypes in multiple tissue sites including the lung and BM.

### 2.3 ABC in unimmunized mice are a heterogenous population of IgD ^+^ and IgD ^−^ ABC

**FIGURE 2:**
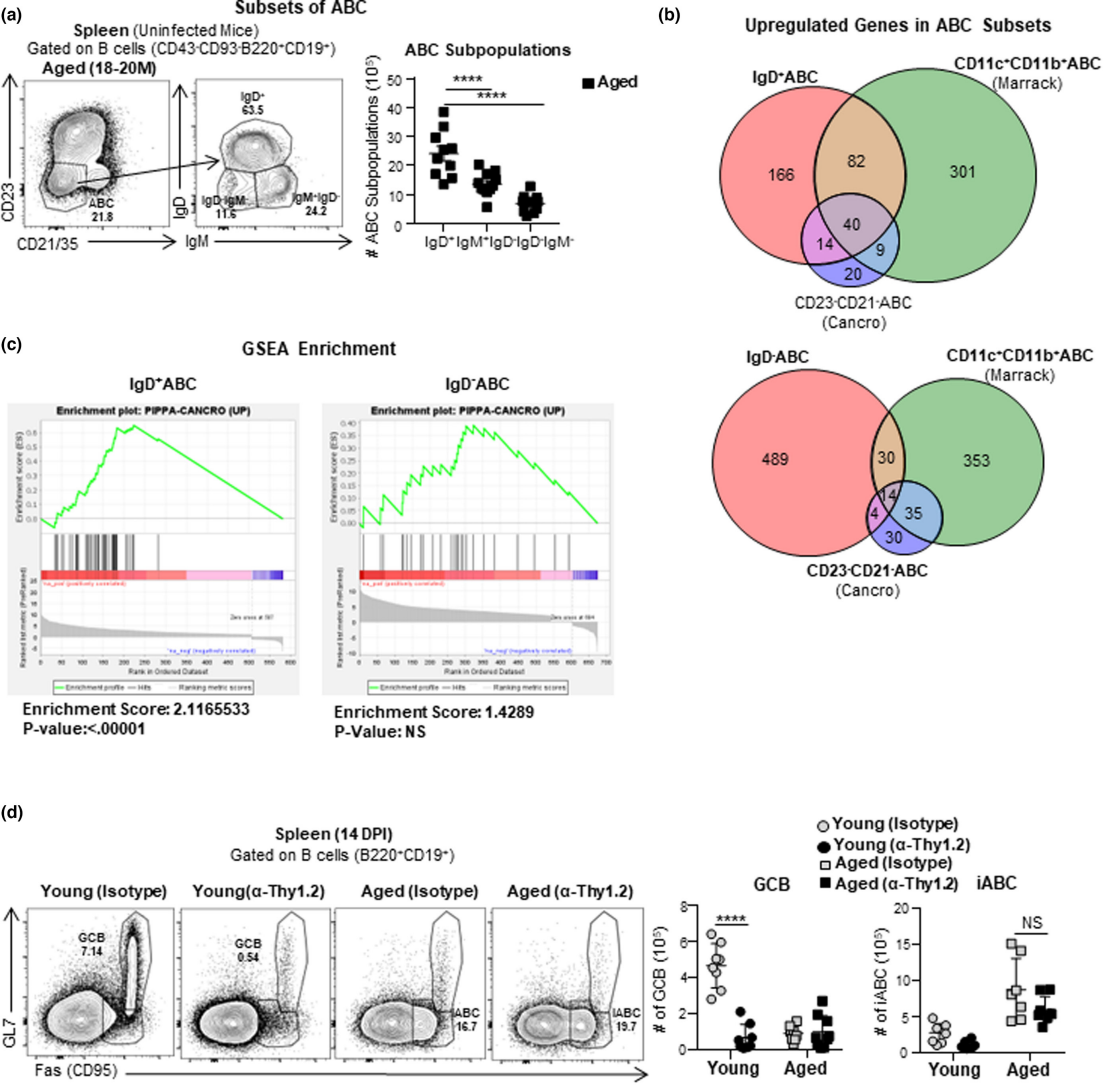
Age‐associated B cells (ABC) subsets and iABC develop independently of T cells. (a) Experimental Design: Uninfected Aged (18–20 M) and young (3–4 M) female B6 mice were sacrificed. CD23^−^CD21^−^ABC subsets in the spleen were analyzed by flow cytometry. Representative FACS plot of gated ABC (CD43^−^CD93^−^B220^+^CD19^+^CD23^−^CD21^−^) (left) and the number IgD^+^, IgM^+^IgD^−^ and IgD^−^IgM^−^ ABC (right) (*n* = 9–12 pooled from 5 to 6 separate experiments) (b, c) Experimental Design: Uninfected Aged (18–20 M) female B6 mice were sacrificed, Spleens were harvested, IgD^+^CD23^−^CD21^−^ABC, IgD^−^CD23^−^CD21^−^ABC and IgD^+^CD23^+^CD21^−^FOB were FACS sorted for RNA sequencing. (b) Venn diagrams showing the fraction of expressed genes shared between IgD^+^ABC and published ABC genes reported by Marrack (Rubtsov et al., 2011) and Cancro (Russell Knode et al., 2017) (top) and the same analysis with IgD^−^ABC (bottom). (c) GSEA plots assessing enrichment of ABC signature genes that are upregulated in sorted IgD^+^ (top) versus IgD^−^ (bottom) ABC (*n* = 2 pooled from 2 separate experiments). (d) Experimental Design: Aged (18–20 M) and young (3–4 M) female B6 mice were treated with anti‐Thy1.2 or isotype control Ab on 0 DPI and 7 DPI, infected with 0.3LD_50_ (25 PFU) PR8 (IAV) and sacrificed at 14 dpi. Spleens were harvested and analyzed for iABC and GCB by flow cytometry. Representative FACS plots and cell numbers of iABC (Fas^+^GL7^−^) and GCB Fas^+^GL7^+^ gated on mature B cells (B220^+^CD19^+^) in anti‐Thy1.2 or isotype control Ab treated aged and young mice in the spleen (*n* = 8–10 pooled from 2 to 3 separate experiments). Error bars represent SEM and statistical significance determined by two‐tailed, unpaired Student's *t*‐test; *p < 0.05; **p < 0.01; ***p < 0.001, ****p < 0.0001.

Some studies have suggested that ABC are memory B cells that accumulate in aged mice (Cancro, 2020; Du et al., 2019; Russell Knode et al., 2017). However, our preliminary data indicated that the largest fraction of ABC express surface IgD (Swain et al., 2017), a phenotype more compatible with a naive B cell population. To evaluate the heterogeneity of the total ABC population in unimmunized aged mice, we analyzed the surface IgD and IgM expression of CD21^−^CD23^−^ABC (Figure 2a) and compared their expression with those of the two conventional naive B cells present: FOB (CD21^+^CD23^+^) and MZB (CD21^+^CD23^−^; Figure S2a). A major population of ABC (about 66%) expressed surface IgD, as did almost all FOB (about 94%) and most MZB (about 84%; Figure S2a). This is consistent with the hypothesis that the population of ABC in unimmunized aged mice is composed mostly of naive B cells. Unlike FOB and MZB, the ABC population also contained a substantial fraction of IgM^+^IgD^−^ B cells (about 21%) and IgM^−^IgD^−^ B cells (about 13%; Figure 2a), indicating the ABC population is heterogenous and suggesting it might contain some Ag‐experienced B cells.

### 2.4 IgD ^+^ ABC share a common gene signature with previously reported ABC

Since the ABC population in unimmunized aged mice contains both IgD^+^ and IgD^−^ B cells, we asked which subpopulation shared more in common with published ABC subsets. We compared IgD^+^ABC and IgD^−^ABC gene expression to the gene expression of ABC found in unimmunized aged mice characterized in previous studies by Cancro (CD21^−^CD23^−^ABC; Russell Knode et al., 2017) and Marrack (CD11c^+^CD11b^+^ABC; Rubtsov et al., 2011; Figure 2b). The IgD^+^ population shared 54 upregulated genes with the Cancro ABC population and 122 upregulated genes with the Marrack ABC population (Figure 2b). In contrast, the IgD^−^ABC subset only shared 18 upregulated genes with the Cancro ABC subset and 44 upregulated genes with the Marrack ABC subset (Figure 2b; Russell Knode et al., 2017; Rubtsov et al., 2011). To confirm that IgD^+^ABC shared a common gene signature with published ABC subsets, we classified the 49 genes that were commonly upregulated by ABC in both the Cancro and Marrack studies (Figure S2c), as a core ABC signature. We then performed a GSEA analysis to check if the core ABC signature was enriched in IgD^+^ and the IgD^−^ABC gene expression. Our analysis showed that the shared ABC signature was significantly enriched in IgD^+^ABC, but not in the IgD^−^ABC population (Figure 2c). Thus, the IgD^+^ABC are most likely related to ABC populations published of Cancro and Marrack.

### 2.5 iABC develop independently of T cells

We know aged mice have a substantially decreased CD4 T helper response (Lefebvre et al., 2016). Since aged mice nonetheless develop a robust iABC in response to influenza (Figure 1), we hypothesized that this subset is less dependent on CD4 T cell help. To further evaluate this possibility, we depleted T cells using anti‐Thy1.2 antibody (Strutt et al., 2012) and then infected young and aged mice with IAV. As expected, Thy1.2 depletion reduced the generation of GCB in young mice compared with isotype control antibody‐treated mice (Figure 2d). In contrast, Thy1.2 depletion did not reduce iABC generation in aged mice (Figure 2d). Therefore, iABC development in aged mice in response to IAV is T‐independent.

### 2.6 IgD ^+^ ABC give rise to iABC

**FIGURE 3:**
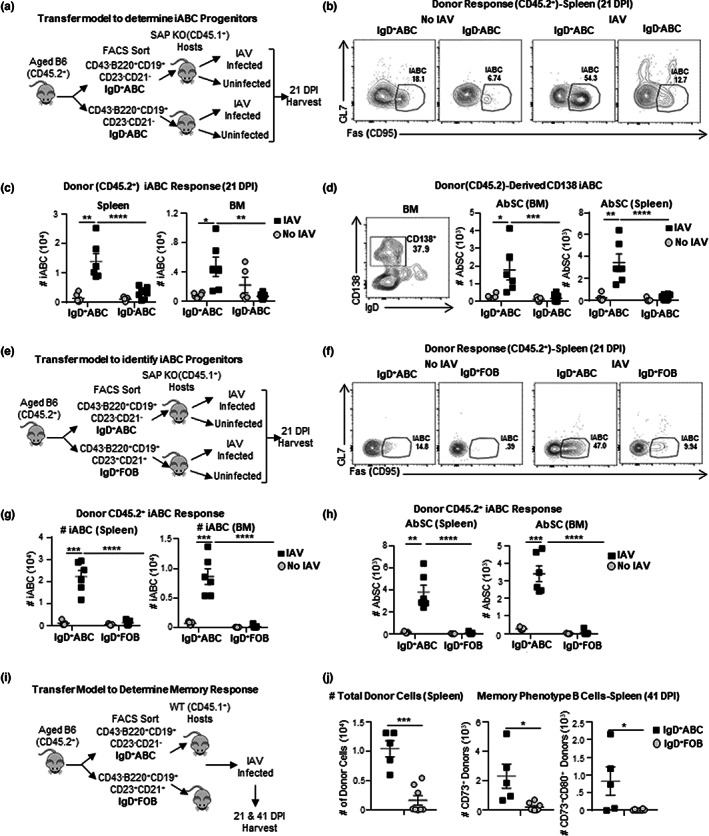
IgD^+^ABC, not IgD^−^ABC or FOB, give rise to Most iABC. (a) Schematic of the transfer model (left): Cells were enriched by positive MACS selection of cells stained with CD43, Ter‐119 and CD23 to remove immature B cells and FOB(CD23^+^CD21^+^). IgD^+^ versus IgD^−^ABC were sorted by flow cytometry, transferred into SAP^−^/^−^.CD45.1 hosts and were infected with 0.4 LD_50_ (33 PFU) PR8 (IAV) or left uninfected (No IAV). Hosts were sacrificed and spleens and BM were harvested at 21 dpi. (b) Representative FACS plots of iABC (Fas^+^GL7^−^) among gated donor B cells in hosts given IgD^+^ and IgD^−^ABC. (c) Cell numbers of total donor (CD45.2) iABC derived from IgD^+^ versus IgD^−^ABC in the spleen and BM of infected versus uninfected SAP^−^/^−^ hosts. (d) Representative FACS plots and cell numbers of total CD138^+^iABC from IgD^+^ versus IgD^−^ABC in the spleen and BM of infected versus uninfected SAP^−^/^−^ hosts. (e) Schematic of the transfer model: CD45.2 spleen cells were enriched by MACS Selection of CD43 and Ter‐119 cells and mature B cells (B220^+^CD19^+^) were sorted into IgD^+^ABC (CD23^−^CD21^−^) and IgD^+^FOB (CD23^+^CD21^+^) by flow cytometry and transferred into CD45.1.SAP^−^/^−^ hosts and either infected with 0.4 LD_50_ (33 PFU) PR8 (IAV) or left uninfected (No IAV). Hosts were sacrificed and spleen and BM were harvested at 21 dpi. Representative FACS plots (f) and cell numbers (g) of total iABC from donor (CD45.2) IgD^+^ABC, and IgD^+^FOB in the spleen and BM of infected versus uninfected CD45.1 SAP^−^/^−^ hosts. (h) Cell numbers of total CD138^+^iABC from IgD^+^ABC and IgD^+^FOB donors of infected versus uninfected CD45.1.SAP^−^/^−^ hosts in the spleen and BM (*n* = 5–6 pooled from 3 to 4 separate experiments; *n* = 5–6 pooled from 3 to 4 separate experiments). (i) Schematic of transfer model and representative FACS plots: IgD^+^ABC (CD21^−^CD23^−^) and IgD^+^FOB (CD23^+^CD21^+^) were sorted from CD45.2 aged mice (18–20 M) by flow cytometry and transferred into wild‐type (CD45.1) hosts and infected with 0.3 LD_50_ PR8 (IAV). Hosts were sacrificed and spleens were harvested at 21 and 41 dpi. (j) cell numbers of recovered donors (CD45.2^+^) cells and memory B cells subsets (CD73^+^, CD73^+^CD80^+^) in IgD^+^ABC and IgD^+^FOB donors in the spleen at 41 DPI (*n* = 5–8 pooled from 2 separate experiments). Error bars represent SEM Statistical significance determined by two‐tailed, unpaired Student's *t*‐test; *p < 0.05; **p < 0.01; ***p < 0.001, ****p < 0.0001.

Since the CD21^−^CD23^−^ABC from aged mice are composed of IgD^+^ and IgD^−^ABC, we asked which subpopulation(s) contains the progenitors that give rise to iABC in response to influenza infection. We hypothesized that if IgD^+^ABC are naive they should have a broad BCR repertoire that would contain a higher fraction of B cells that could respond to IAV and give rise to more iABC. We developed an adoptive transfer system using SAP^−/−^ hosts that are deficient in T cell help. SAP^−/−^ mice do not generate T_FH_ responses (Kamperschroer et al., 2006, 2008), and thus, mimic the aging host environment where few T_FH_ develop in response to influenza infection (Lefebvre et al., 2016). We sorted CD45.2^+^, aged IgD^+^ABC and IgD^−^ABC (Figure S3a) and transferred them into young allotype‐marked (CD45.1) SAP^−/−^ hosts and infected with IAV (Figure 3a). Since ABC have been implicated in autoimmunity (Phalke & Marrack, 2018), we also transferred each subset to uninfected (no IAV) SAP^−/−^ hosts to determine if indeed the responses we generate were driven by IAV infection (Figure 3a). Analysis of donor populations at 21 dpi, identified by CD45.2, revealed that donor IgD^+^ABC gave rise to an iABC population (Fas^+^GL7^−^) in both the spleen and BM. In contrast, the IgD^−^ABC subset gave rise to only a very small iABC response (Figure 3b,c). As expected, iABC were generated in IAV‐infected, but not in uninfected mice (Figure 3b,c). There was a substantial population of donor cells that retained the naive phenotype, expressing neither GL7 nor Fas (Figure 3b) even in hosts that were infected, which is expected, since we transferred a population of polyclonal naive ABC, of which only a very small fraction are expected to have a BCR that recognizes IAV.

To determine if the iABC derived from the transferred IgD^+^ABC donors become AbSC, we stained for CD138 (Nutt et al., 2015) and analyzed by FACS. Following immunization, most of the iABC (80%) derived from the transferred IgD^+^ABC, had lost IgD expression, and a fraction expressed CD138^+^, indicating they were AbSC, in the spleen (28%) and BM (38%; Figure 3d). This data indicates that in the aged, IgD^+^ABC are the B cell precursors with the highest potential to differentiate into the iABC and AbSC in response to a primary infection.

### 2.7 IgD ^+^ ABC, not FOB, are the progenitors of iABC

Aged mice still maintain a substantial population of FOB and a small one of MZB cells (Hao et al., 2011), either of which could potentially give rise to iABC. Studies show that MZB responses are largely impaired due to several mechanisms in the aged (Birjandi et al., 2011; Turner & Mabbott, 2017), making them unlikely to be precursors of iABC. However, if some FOB can respond in the aged, they might contribute to the AbSC response. We isolated equal numbers of IgD^+^ABC and IgD^+^FOB, from aged, uninfected B6 mice, and transferred them into separate groups of infected or uninfected SAP^−/−^ CD45.1 hosts (Figures 3e and S3c). After 21 dpi, we assessed the number of iABC formed from each of the donor subsets, in the spleen and BM (Figures 3f,g and S3d). Hosts that received IgD^+^ABC donor cells developed a substantial iABC response in spleen and BM, while FOB generated only background levels of iABC (Figure 3f,g). None of the uninfected hosts developed significant iABC. Moreover, CD138^+^iABC developed from IgD^+^ABC, but not from FOB in the spleen and in BM (Figure 3h). Thus, IgD^+^ABC, but not IgD^+^FOB, gave rise to iABC that become AbSC in response to IAV infection.

In the above experiment in SAP KO hosts, the IgD^+^FOB did not give rise to iABC or other strong responses but it is possible aged FOB could give effector B cell responses in a host with young WT CD4 T cells. Therefore, we sorted IgD^+^ABC and IgD^+^FOB from aged mice (CD45.2), transferred each into young WT hosts (CD45.1) and infected these hosts with IAV (Figure 3i). We analyzed iABC and GCB generation at 21 dpi. Similar to results in SAP^−/−^ hosts, donor IgD^+^ABC gave rise to many more iABC (Fas^+^GL7^−^) than aged donor FOB in the WT hosts (Figure S4a). Next, we analyzed the expression of GL7, Fas, CD80, CD11b, CD11c, and T‐bet of total donor‐derived (CD45.2) cells at 21 dpi. Although donor FOB made very few iABC, we wanted to confirm this using additional published iABC markers. As expected (Figure 1), the total population of IgD^+^ABC donor‐derived effectors expressed high levels of Fas but not GL7, and clearly expressed more of the previously published iABC markers CD80, CD11b, CD11c, and T‐bet compared with FOB donors (Figure S4b) confirming that IgD^+^ABC are the progenitors of iABC. These results, combined with those in SAP^−/−^ hosts and using multiple ABC markers, strongly support the concept that in the aged mice (18–20 M), responses to IAV predominantly come from IgD^+^ABC progenitors.

### 2.8 Responding IgD ^+^ ABC, not FOB become memory B cells

We asked if IgD^+^ABC could give rise to memory responses during IAV infection. Using the transfer model described above (Figure 3i), we quantified memory B cell responses from IgD^+^ABC donors, at 41 dpi in the spleen. We recovered a much higher number of donor cells derived from IgD^+^ABC precursors than from FOB (Figure 3j) and these included higher numbers of CD73^+^ cells and CD73^+^CD80^+^ cells, that have been classically used to define memory B cells (Johnson et al., 2020; Onodera et al., 2012) indicating that IgD^+^ABC (Figures 3j and S4c) can give rise to memory B cells. Thus, IgD^+^ABC can differentiate into memory phenotype B cells in response to influenza infection, suggesting that they are likely to have potential to confer long‐term protection in addition to Ab responses.

### 2.9 ABC accumulation is independent of commensal bacteria and foreign Ag

**FIGURE 4:**
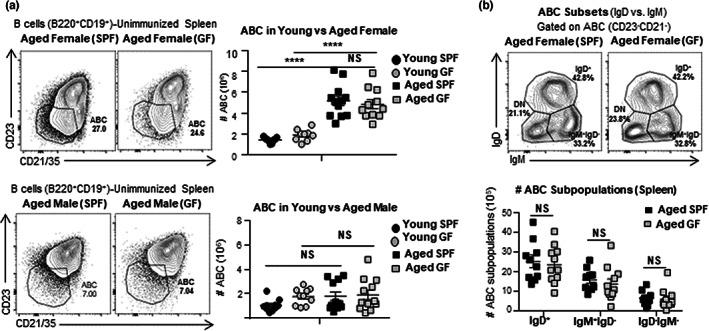
ABC subsets accumulate with age in germ‐free (GF) and specific pathogen free (SPF) mice. (a) Experimental Design: Unimmunized specific‐pathogen free (SPF) and germ‐free (GF), aged (22–24 M) and young (3–4 M) female and male B6 mice were sacrificed. Spleens were harvested and analyzed by flow cytometry. Representative FACS plots and cell numbers of total ABC (CD21^−^CD23^−^) in aged SPF versus GF B6 mice (b) Representative FACS plots and cell numbers IgD^+^, IgM^+^IgD^−^, and IgD^−^IgM^−^ABC subsets in aged SPF versus GF female B6 mice (*n* = 9–12 pooled from 7 to 8 separate experiments) Error bars represent SEM Statistical significance determined by two‐tailed, unpaired Student's *t*‐test; *p < 0.05; **p < 0.01; ***p < 0.001, ****p < 0.0001.

Little is known of the origins of the progenitor ABC that accumulate with aging. Previous studies suggested that Ag exposure plays a role in the generation of CD21^−^CD23^−^ABC (Cancro, 2020; Russell Knode et al., 2017), but those studies did not separately evaluate the IgD^+^ progenitor subset and did not determine if Ag exposure was due to foreign or self Ag. We speculated that the development of the IgD^+^ABC subset with age would not require foreign Ag stimulation, though we considered it might have a role in generation of the IgD^−^ subsets. To address the role of foreign and commensal Ag exposure, we compared ABC from our aged specific pathogen free (SPF) mice that contain Ag from commensal organisms and are exposed to foreign Ag from the environment, with aged germ‐free (GF) mice that lack commensal microbes and are isolated throughout life to prevent exposure to environmental Ag. Aged female SPF and GF mice had equivalent high proportions and absolute numbers of splenic ABC (Figure 4a). Like young SPF mice, young GF mice did not develop a distinct ABC population (Figures 4a and S5a,b). We used surface staining to assess IgD^+^ and IgD^−^ABC subsets within the ABC. The percentage and numbers of ABC that are IgD^+^, IgD^−^IgM^+^, and IgD^−^IgM^−^ were comparable in SPF and GF female mice (Figures 4b and S5c). In both SPF and GF aged mice, the IgD^+^ population was the largest population. We also compared the numbers of ABC in male and female mice. Aged male mice, both SPF and GF, had lower proportions of ABC compared with females, consistent with earlier indications that female mice accumulate more ABC with age (Hao et al., 2011; Russell Knode et al., 2017; Figure 4a). Thus, with age, accumulation of ABC occurs independent of exposure to foreign or commensal Ag and is most prevalent in female mice. This supports the concept that an intrinsic developmental program drives the development of ABC rather than extrinsic factors such as environmental or commensal Ag stimulation.

### 2.10 Development of ABC is independent of TLR7 and TLR9

**FIGURE 5:**
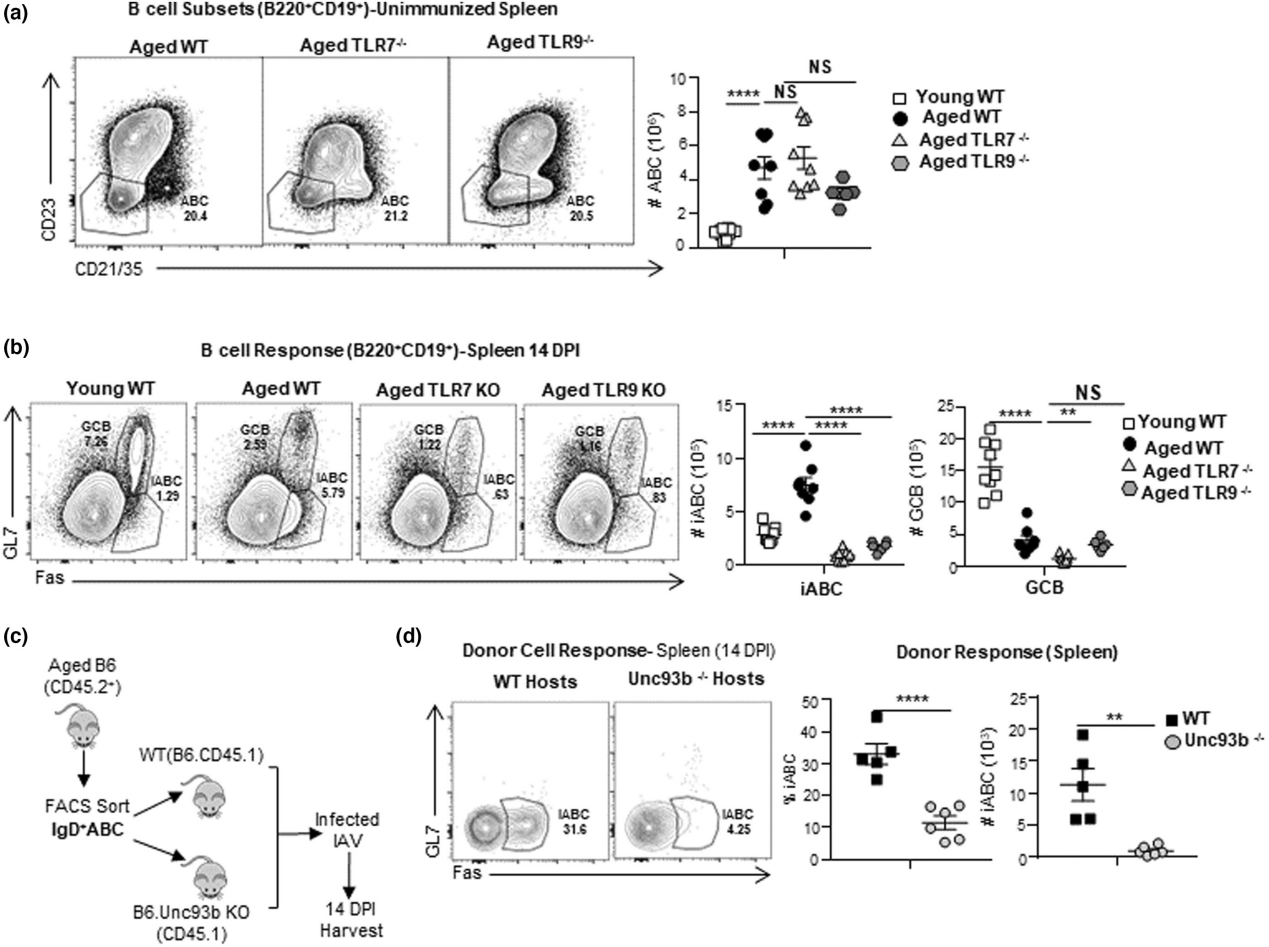
Age‐associated B cells (ABC) develop independent of endosomal TLR signals, while iABC generation from ABC requires extrinsic endosomal TLR signaling. (a) Experimental Design: Unimmunized Young WT, Aged WT, TLR7^−^/^−^and TLR9^−^/^−^ female BALB/c mice were sacrificed and B cells populations analyzed in the spleen by flow cytometry. Representative FACS plots and cell numbers of total CD21^−^CD23^−^ABC in young WT and aged WT, TLR7^−^/^−^ and TLR9^−^/^−^ female BALB/c mice (*n* = 5–11 pooled from 2 to 3 separate experiments). (b) Experimental Design: Young WT and aged WT, TLR7^−^/^−^ and TLR9^−^/^−^ female BALB/c mice were infected with 0.4LD_50_ (33 PFU) PR8 (IAV) and sacrificed at 14 dpi. Spleens were harvested and analyzed by flow cytometry. Representative FACS plots and cell numbers of total iABC (Fas^+^GL7^−^) and GCB (Fas^+^GL7^+^) found in young WT and aged WT, TLR7^−^/^−^ and TLR9^−^/^−^ female BALB/c mice (*n* = 6–9 pooled from 2 to 3 separate experiments). (c) Schematic of the transfer model: IgD^+^ABC (CD45.2) from aged B6 mice were isolated by sorting as in previous experiments and transferred into B6.CD45.1 WT or B6.CD45.1.Unc93b^−^/^−^ hosts, that were infected with 0.4 LD_50_ (33 PFU) PR8 (IAV). Hosts were sacrificed and spleens were harvested at 14 dpi. (d) Representative FACS plots, percentage, and cell numbers of total iABC from donor CD45.2 IgD^+^ABC in the spleen of infected CD45.1 WT versus Unc93b^−/−^ hosts (*n* = 5–6 pooled from 2 to 3 separate experiments). Error bars represent SEM Statistical significance determined by two‐tailed, unpaired Student's *t*‐test; *p < 0.05; **p < 0.01; ***p < 0.001, ****p < 0.0001. NS = not significant.

Previous studies have suggested that TLR7 plays a key role in the development of CD11c^+^ABC in autoimmune models and unimmunized aged mice (Naradikian et al., 2016; Rubtsov et al., 2011, 2013). To ask if progenitor ABC required TLR7 and TLR9 for their development, we examined the ABC populations that developed in aged TLR7^−/−^ and TLR9^−/−^ BALB/c mice. ABC development was not impaired in either aged TLR7^−/−^ or aged TLR9^−/−^ mice, and was comparable to that in wild‐type (WT) aged BALB/c mice (Figures 5a and S5d). Additionally, the percentage and numbers IgD^+^, IgD^−^IgM^+^, and IgD^−^IgM^−^ABC were comparable in the aged WT, TLR7^−/−^ or aged TLR9^−/−^ mice (Figure S5e). Thus, neither TLR7 nor TLR9 signaling is required for ABC accumulation with age. This further supports the concept that the ABC and its and subsets develop by an intrinsic program associated with aging, independent of external signals from pathogen recognition that trigger TLR7 and TLR9 pathways.

### 2.11 IgD ^+^ ABC require cell‐extrinsic endosomal TLR7 and TLR9 signals to generate iABC

Signaling through endosomal toll‐like receptors (TLRs) and other pathogen recognition (PR) receptors has been shown to markedly enhance conventional anti‐viral responses of FOB in young mice (Iwasaki & Medzhitov, 2015; Rookhuizen & DeFranco, 2014). In vitro TLR7 and TLR9 stimulation are needed to activate CD23^−^CD21^−^ABC isolated from unimmunized aged mice (Hao et al., 2011) and drive autoimmune responses mediated by ABC‐like cells (Phalke & Marrack, 2018). To determine what role TLR recognition pathways play in the generation of the iABC response to influenza infection, we infected TLR7 and TLR9 deficient aged mice and compared their responses to WT aged and young mice at 14 dpi. Neither TLR7^−/−^ nor TLR9^−/−^ mice developed iABC (Figures 5b and S5f). Very few GCB developed in aged mice compared with young WT (Figure 5b). Thus, unlike the progenitor ABC which develop independently of both foreign Ag and TLR7 and TLR9 pathways, the iABC response to influenza infection is highly dependent on both endosomal TLR7 and TLR9 pathways.

Most studies have focused on the cell‐intrinsic role of TLR signals in ABC activation (Hao et al., 2011; Jenks et al., 2018; Rubtsov et al., 2011, 2013; Teichmann et al., 2013). However, other studies have shown that TLR‐dependent activation of dendritic cells can regulate autoreactive B cell response (Ols et al., 2016; Teichmann et al., 2013). Whether the ABC response depends only on direct TLR activation of the responding B cells (cell‐intrinsic) or if it also depends on TLR activation of non‐ABC cells (cell‐extrinsic) remains unclear. To evaluate this, we transferred IgD^+^ABC from WT aged B6 mice into B6.Unc93b^−/−^ recipients (Figure 5c). Unc93b is a chaperone protein required for the trafficking of TLR3, TLR7, and TLR9 receptors to the endocytic compartments where they engage their ligands, and thus, Unc93‐deficient mice cannot mount effective endosomal TLR responses (Pelka et al., 2018). The recipients were challenged with IAV, and we analyzed iABC responses from donor cells at 14 dpi (Figure 5d). Wild‐type IgD^+^ABC donors developed fewer iABC in Unc93^−/−^ than WT control hosts. This indicates that iABC generation from ABC progenitors requires extrinsic activation of TLR in host cells.

### 2.12 ABC responses to IAV result in neutralizing anti‐influenza Ab

**FIGURE 6:**
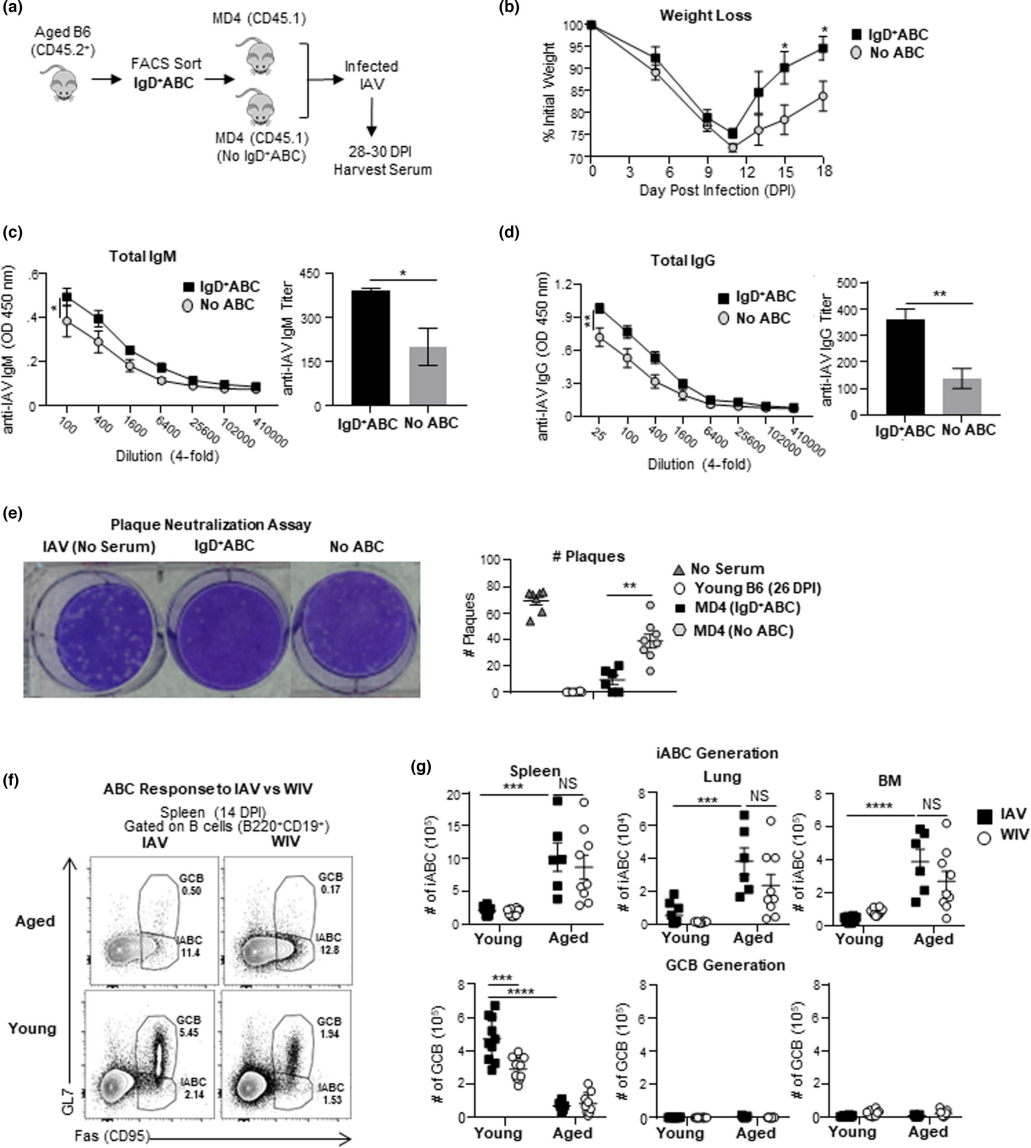
IgD^+^ABC produce neutralizing anti‐IAV Ab and inactivated IAV immunization generates iABC. (a) IgD^+^ABC (CD45.2) were sorted by flow cytometry and transferred into CD45.1 MD4 hosts and infected with 0.3 LD_50_ (25 PFU) PR8 (IAV). Hosts were sacrificed and serum was collected at 28–30 dpi (b) Kinetics (0–18 dpi) of weight loss between MD4 hosts with IgD^+^ABC and without IgD^+^ABC ELISA were performed with 28–30 dpi. Serum was collected from individual mice in both groups. (c) The titer of anti‐IAV‐specific IgM Ab was determined by ELISA. OD curves are shown over the titrated range (left) and the titer of anti‐IAV Ab (right) (d) The titer of anti‐IAV‐specific total IgG Ab OD curves is shown over the titrated range (left) and the titer of anti‐IAV Ab (right) (e) Plaque Neutralization Assay: using serum from the MD4 hosts with transferred IgD^+^ABC versus no ABC (*n* = 5–6 pooled from 2 to 3 separate experiments) Shown is an example of the plaque assay (left) and all the data from individual serum samples (right). (f) Experimental Design: Aged and young B6 mice were infected with 0.3 LD_50_ (25 PFU) PR8 (IAV) i.n. or treated with 5 μg whole inactivated PR8 (WIV) i.v. and sacrificed at 14 dpi. Representative FACS plots showing iABC and GCB responses in the spleen of aged and young B6 mice (g) The iABC (top) and GCB response (bottom) responses in spleen, lungs, and BM from individual mice in each group and is shown. Error bars represent SEM. Statistical significance determined by two‐tailed, unpaired Student's *t*‐test; *p < 0.05; **p < 0.01; ***p < 0.001, ****p < 0.0001.

In Figure 1, we showed that iABC are the major responding population and become AbSC to primary immune response to influenza infection in the aged. To evaluate whether ABC‐derived Ab can combat influenza infection in an adoptive transfer model, we needed a host with very limited B cell responses to influenza. The SAP^−/−^ mice we used as hosts previously (Figure 3), still have B cells that can mount T_FH_‐independent antibody responses (Kamperschroer et al., 2006), so are not useful for assessing ABC‐derived protection. We choose MD4 BCR transgenic mice whose B cells do not respond to IAV, because the MD4 receptor, specific for hen egg lysozyme (HEL) (Bortnick et al., 2012) We transferred sorted IgD^+^ABC into MD4 hosts, infected them with IAV and collected the serum at 28–30 dpi. MD4 hosts, not given ABC cells prior to infection, were used as negative controls (Figure 6a). MD4 with transferred IgD^+^ABC, recovered more weight than control MD4 hosts without transferred cells (Figure 6b). At 28–30 dpi, we collected serum to evaluate Ab levels and determine their ability to neutralize virus. MD4 hosts with ABC had significantly higher titers of anti‐influenza IgM and IgG in their serum compared with MD4 hosts without ABC (Figure 6c,d). We used a plaque neutralization assay to determine if the serum Ab could neutralize influenza A virus (IAV). The serum from MD4 hosts with transferred IgD^+^ABC, neutralized IAV preventing plaque formation (Figure 6e), while that from hosts without transferred ABC failed to do so. This suggests IAV‐induced iABC make IAV‐specific Ab responses, which can neutralize virus and hence have the potential to provide significant protection from influenza infection in aged animals.

### 2.13 ABC also generate an iABC response to inactivated influenza immunization

Compared with younger adults, the aged respond poorly to many current influenza vaccines due to declining CD4 and CD8 T cell responses as well as B cell responses (Dugan et al., 2020; McElhaney et al., 2016). We showed that ABC can mount a T‐cell independent iABC response to live IAV in aged mice (Figure 2) and that this depends on endosomal TLR signaling from host cells (Figure 5). Whole inactivated influenza virus (WIV) contains all influenza components including many B cell epitopes, and studies indicate they can stimulate TLR7 and TLR9 (Fink et al., 2018; Onodera et al., 2016). We compared the ability of WIV immunization and live IAV infection, to stimulate iABC generation in intact aged and young mice, as in our earlier studies (Brahmakshatriya et al., 2017). We examined B cell responses at 14 dpi, so we could assess both iABC and GCB development in spleen, lung, and BM. In aged mice, both WIV immunization and IAV infection generated equivalent numbers of iABC in the spleen, lung, and BM. As expected in young mice, neither immunization nor infection generated iABC, and IAV generated a greater GCB response than WIV in the spleen (Figure 6f,g). These results suggest that WIV can provide sufficient signals from Ag and TLR to drive ABC to become iABC and provide proof of principle that vaccine approaches like WIV, may succeed in harnessing ABC responses in the aged.

## 3 DISCUSSION

The inability to effectively vaccinate older individuals against life‐threatening pulmonary infections is a major public health concern, so determining how to induce protective immune response in the eldery critically important. We showed that aged mice (18–20 M) do not mount an effective GCB response following primary influenza infection, but instead generate a novel B cell response, consisting of a prominent Fas^+^GL7^−^ (iABC) population that also express previously published ABC markers (T‐bet, CD11c, and CD11b). The iABC are derived from a naive subset of IgD^+^ABC. These progenitors can become memory B cells and AbSC that produce IgM and some IgG Ab that is capable of neutralizing IAV. Importantly, immunization with WIV as a vaccine, elicited a comparable iABC response in aged mice as live infection. Thus, it seems likely that WIV provides high enough Ag and PR signals to generate iABC in the aged. Inactivated vaccines have been successfully used in the aged for decades and thus are a feasible and safe approach to induce immunity in the aged. The success of WIV immunization may provide an indication of approaches that might that can safely harness ABC responses as an alternate source of AbSC that protect elderly populations from new pathogens or strains.

We found that the iABC generated after influenza infection mostly expressed IgM and were found in the spleen, lung, and BM. This distribution resembles that seen for other T‐independent responses of B1 and marginal zone B cells earlier in life, which mostly produce extrafollicular IgM^+^ Ab responses and also generate memory B cells (Allman et al., 2019; Cerutti et al., 2013). Studies show that IgM^+^ memory B cells have a diverse repertoire and contribute to long‐lasting durable protection (Allman et al., 2019; Bohannon et al., 2016). In young mice, some effector B cells made in a primary response migrate to the lung early following infection, are also GC‐independent, predominantly IgM, and are especially protective against respiratory infection (Onodera et al., 2012). In young mice, IgM Ab can neutralize influenza virus and they are maintained over 18 months in infected and immunized mice, predicting that they should be capable of providing durable protection (Bohannon et al., 2016; Skountzou et al., 2014). We speculate that in the aged, iABC that are predominantly IgM^+^, could be a source of Ab‐mediated protection following exposure to novel or emerging infections, replacing the impaired conventional B cell response.

The iABC generated by infection in aged mice contained a small number of IgG2b^+^AbSC in the spleen, lung, and BM and some IgA^+^AbSC cells in the lung and BM. This suggests that the iABC response drives a significant, though modest, level of isotype‐switching. In young mice, infection generates high levels of IgG2b and IgA, which contribute to Ab‐medicated protection (Abreu et al., 2020; Miyauchi et al., 2016). Young IgG2b Ab generation is independent of T_FH,_ but still depends on IFNγ secreted by T_H1_ cells (Miyauchi et al., 2016). We previously found that when we provided TLR‐activated antigen‐presenting cells (APC) to aged mice, T_H1_ effectors and T_FH_ responses were largely restored and this resulted in higher total IgG titers, including increased IgG2b, in aged hosts (Brahmakshatriya et al., 2017). Given our findings here, it is likely that much of that response may have been provided by ABC becoming iABC, and thus that strategies designed to enhance isotype‐switching in iABC could potentially be developed and provide higher IgG responses as well in the aged.

Most previous studies have not considered CD21^−^CD23^−^ABC as a heterogeneous population at different stages of development, some but not all of which are likely to be previously Ag‐experienced (Hao et al., 2011; Russell Knode et al., 2017). For instance, Cancro's studies suggested that Ag presentation drives generation or development of CD21^−^CD23^−^ABC in unimmunized aged mice and concluded ABC are memory cells (Cancro, 2020; Russell Knode et al., 2017). Most ABC studied in humans are likely memory cells and those in autoimmune conditions are likely self Ag‐driven (Kugler‐Umana et al., 2020). However, we found that the ABC population in aged unimmunized SPF and GF mice, lacking most potential pathogen exposure, is composed of three subpopulations: a predominant IgD^+^ subset and a smaller IgD^−^ subset, consisting of IgM^+^ and IgM^−^ B cells (Figure 2). Thus, in GF mice, most IgD^+^ and IgD^−^ABC developed without foreign Ag exposure. It remains unclear whether the development of the IgD^−^ABC subsets during aging, depends on self‐Ag recognition though there are studies indicating that some of the total CD21^−^CD23^−^ABC in unimmunized SPF mice have undergone some affinity maturation compared with FOB (Russell Knode et al., 2017), suggesting that they may contain some Ag‐experienced B cells. We suggest that the IgD^−^ABC subset may account for those results. Our data here shows that IgD^+^ABC are the progenitors of a predominant aged B cell response against influenza infection, suggests that IgD^+^ABC express a broad BCR repertoire that includes a cohort that recognizes influenza and can respond to infection in aged mice.

Our studies in SAP^−/−^ hosts (T_FH_ deficient) and T cell‐depleted aged mice show that iABC generation from IgD^+^ABC does not depend on CD4 T cell help or T_FH_ (Figures 2 and 3). The T‐cell independence of this naive ABC response is consistent with the known reduction of naive CD4 T cell response, including T_FH_, to infection and to vaccines with age (Haynes & Swain, 2006). In contrast, the autoimmune ABC response has been reported to require IFNγ and IL‐21 produced by effector CD4 T cells (Manni et al., 2018; Naradikian et al., 2016; Wang et al., 2018), which contrasts with the T‐independent generation of iABC. However, both the iABC population here (Figure 1) and the published autoimmune ABC (Kugler‐Umana et al., 2020) express CD11c and T‐bet, classic ABC markers as well as CD11b and CD80 (Figure S4b). This raises the possibility that ABC responses found in these different contexts are driven by distinct pathways and that CD11c and T‐bet may be expressed by distinct effector ABC subsets. The autoimmune ABC response may require CD4 T cell help, unlike anti‐pathogen iABC responses, which we postulate depend on higher pathogen and Ag‐presentation signals. Further definition of the strength and duration of signals needed to drive ABC autoimmune versus protective pathways in mice of different ages and sexes, will clearly be needed to define the full potential of ABC and to develop safe but effective vaccines that harness the protective IgD^+^ABC in the aged.

T‐cell independent B cell responses rely more on PR pathways for their generation (Bortnick et al., 2012). Many studies have shown that cell‐intrinsic TLR signaling plays an important role in GCB responses in somatic hypermutation and isotype class switching and act in conjunction with T_FH_ help (Browne, 2012). Early activation of extrafollicular autoimmune B cells in SLE, which includes different ABC subsets (Jenks et al., 2018; Rubtsov et al., 2011), depends on cell‐intrinsic TLR7 signaling (Fillatreau et al., 2021; Teichmann et al., 2013). We asked whether endosomal viral‐sensing TLR signals in the B cells or in other innate cells, known to be strongly induced by influenza RNA, are required for iABC generation We found that iABC did not develop in response to influenza infection, in either aged TLR7^−/−^ or TLR9^−/−^ mice, indicating that both TLR are required for iABC generation (Figure 5). Additionally, we found iABC development from IgD^+^ABC depends on cell‐extrinsic TLR pathways, indicating non‐ABC intrinsic pathways also play key roles in iABC generation (Figure 5) (Pelka et al., 2018). We postulate that high dependence of the ABC response on both TLR7 and TLR9, and on cell‐extrinsic endosomal TLR expression, limits ABC responses to those stimulated by pathogens which provide very strong stimulation of viral‐sensing PR pathways (Kugler‐Umana et al., 2020; Swain et al., 2021).

The pathways of development of IgD^+^ABC in unimmunized mice remains unclear. One hypothesis is that IgD^+^ABC are a naive subset that develops as part of an intrinsic age‐associated developmental pathway (Swain et al., 2021). We postulate this has been selected by evolution to limit autoimmunity but still allow responses to replicating pathogens that provide high sustained Ag and abundant PR signals. Another possibility is that with increasing age, innate immune cells undergo changes that lead to a shift in homeostatic factors that favor progenitor ABC development.

Since IgD^+^ABC become AbSC and memory B cells (Figure 3), we tested whether the ABC‐derived Ab provide some level of protection against influenza (Figure 6). We transferred 10^6^ polyclonal naive IgD^+^ABC cells, which represents a small proportion of the in‐situ aged ABC population into MD4 hosts. We show that MD4 hosts with transferred IgD^+^ABC make increased IAV‐specific IgM and IgG serum Ab titers compared with host without ABC and these Ab can neutralize IAV in a plaque assay (Figure 6). The presence of ABC in IAV‐infected MD4 mice also induced faster recovery from weight loss. Thus, the increase in anti‐IAV serum Ab after transfer of ABC has a modest but significant effect on IAV clearance. Therefore, we suggest that ABC responses against influenza are a critical part of protection against novel infectious pathogens in the aged. We also show that IgD^+^ABC can become memory in the spleen (Figure 3j). If they become long‐lived influenza‐specific LLPC in the BM and/or memory B cells in the lung, which we have not yet established, they could provide substantial local and systemic protection.

We found that in aged mice, whole inactivated influenza vaccine (WIV) efficiently generates iABC in the spleen, lung, and BM (Figure 6). This raises the possibility that some vaccine strategies may provide sufficient Ag and PR signals to generatate iABC. However, whether WIV alone provides enough signals for iABC to become AbSC and memory B cells remains unclear. Several studies have shown that immunized aged mice are better protected against infection than unmmunized mice (Baldwin et al., 2018; Cookenham et al., 2020; Petsch et al., 2012; Ross et al., 2019). Administration of two doses of a split H1NI vaccine (Baldwin et al., 2018) or recombinant nucleoprotein vaccine (Cookenham et al., 2020) combined with a TLR4 adjuvant increased protection against lethal H1N1 challenge in aged mice over vaccine alone. Other vaccine platforms such as nanovax that provide increased PR signals, drive an increase of anti‐HA Ab titers in aged mice (Ross et al., 2019). Similar to these adjuvanted vaccines, one dose of influenza mRNA vaccine, which induced TLR7‐mediated adaptive responses, protected aged mice from lethal influenza challenge (Petsch et al., 2012). Thus, effective vaccine‐generated immunity seems to correlate with providing increased levels of PR signals in the aged. Since iABC is the major responding B cell subset in aged mice, we propose that protection from lethal challenge quantified in these studies depends on this subset. We suggest that readouts of iABC induction should be studied as a correlate of protection to target more effective vaccines for the aged.

Our data suggests that ABC progenitors develop during aging as an adaptation to mount immunity against novel pathogens via iABC effector responses. In contrast to other naive T and B immune responses whose function declines drastically with age, the ABC response seems inherently specialized to respond in an aged environment. Defining each of the factors required for protective ABC responses should lead to important insights into how to formulate and deliver vaccines to generate ABC‐mediated protection and improve in anti‐pathogen immunity in the highly vulnerable aged populations.

## 4 MATERIALS AND METHODS

### 4.1 Mice

Young C57BL/6 (B6), BALB/c, and B6.CD45.1 mice were obtained from the Jackson Laboratory. Aged (8–12 weeks) C57BL/6 (B6), BALB/c mice were obtained from the National Institute of Aging (NIA). SAP^−/−^.CD45.1 mice and maintained in standard specific pathogen free (SPF) housing at the Umass Chan facility. MD4. Ig‐tg mice were obtained from Dr. Andrea Reboldi, bred with B6.CD45.1 mice and maintained in standard specific pathogen free (SPF) housing at the Umass Chan facility. BALB/c.TLR7^−/−^, BALB/c TLR9^−/−^, and Unc93b^−/−^ mice were obtained from Dr. Ann Rothstein and these strains were bred and maintained at the Umass Chan animal facility. Young and aged germ‐free C57BL/6 mice were bred and maintained in the Gnotobiotic Core at the College of Veterinary Medicine, North Carolina State University. Germ‐free mice were housed in flexible film isolators and provided with autoclaved food and water. Germ‐free status was evaluated at least once a month by culturing stool samples in aerobic and anaerobic conditions. Aged SPF and GF mice were at least 72 weeks old and young mice were at least 8 weeks old prior to use. Animal use protocols were approved by the IACUCs at Umass Chan and at North Carolina State University.

### 4.2 Virus stocks, infections, and immunizations

Influenza A viruses (IAV) A/Puerto Rico/8/34 (PR8), originally from St. Jude Children's Hospital, kindly provided by Dr. Peter Doherty, were grown and maintained at the Trudeau Institute. IIV (WIV): Formalin‐inactivated influenza vaccine (A/PR/8/34 [H1N1]) was purchased from Charles River Laboratories (material no. 10100782) and used at a dose of 5 μg intravenously as in our previous studies (Xia et al., 2020). Mice were anesthetized with isoflurane (Piramal Healthcare) and were infected intranasally with influenza virus corresponding to a 0.3–0.4 LD_50_ (25–33 PFU or 1500–2000 EID_50_) dose of IAV in 50 μl of PBS.

### 4.3 Adoptive B cell transfer and T cell depletion

Aged splenocytes were depleted of RBC and CD19^+^B220^+^ B cells were enriched by positive selection of CD43 and Ter‐119 cells and LD MACS columns (Miltenyi Biotec). CD43^−^Ter‐119^−^ splenocytes were stained with FITC anti‐B220 (Biolegend) BV421 anti‐CD19 (Biolegend), PerCP Cy5.5 anti‐CD23 (eBiosciences), APC‐fluor 780 anti‐CD21/35 (eBioscience), PE‐Cy7 anti‐IgD (Biolegend), APC anti‐CD43 (eBioscience), and APC anti‐CD93 (AA4.1; eBioscience). CD23^+^(FOB), IgD^+^ and IgD^−^CD21^−^CD23^−^ (ABC) lymphocytes were sorted on a BD FACS Aria III (BD Biosciences) at Umass Chan Flowcore. Flow cytometric sorting yielded enrichments between 90% and 95% purity for each subset. 10^6^ cells of each sorted subset were transferred intravenous into SAP^−/−^ or MD4 transgenic hosts were sacrificed, and spleen, BM, and serum were harvested at 21–30 DPI.

In some experiments, young and aged mice were treated i.p. with 250 μg of either anti‐Thy1.2‐depleting antibody (30‐H12; Bio X Cell) or with an isotype control on 0 DPI and 7 DPI (Strutt et al., 2012). Anti‐Thy1.2 or isotype‐control‐treated mice were infected with 0.3LD_50_ (25 PFU) dose of IAV, sacrificed at 14 dpi and spleens were harvested to analyze B cell responses.

#### 4.3.1 RNA isolation and sequencing

2 × 10^6^ CD23^+^ (FOB), IgD^+^, and IgD^−^ CD21^−^CD23^−^ (ABC) lymphocytes were isolated as described above and RNA was extracted using RNAeasy plus Mini kit (Qiagen). RNA quality was checked by mRNA fragment analysis performed by the Molecular Biology Core are UMMS. RNA‐sequencing were sent to Beijing Genomics Institute (BGI), Shenzhen, China, for 50 bp single‐end sequencing by BGISEQ‐500 sequencer. Oligo (dT) magnetic beads were used to select mRNA with polyA tail or hybridized the rRNA with DNA probe and digested the DNA/RNA hybrid strand, followed by DNase I reaction to remove DNA probe. After DNA removal, the target RNA was fragmented and reverse transcripted to the double‐strand cDNA (dscDNA) by N6 random primer. The dscDNA was end repaired with phosphate at 5′ end and stickiness “A” at 3′ end, then ligated and adapted with stickiness T at 3′ end to the dscDNA. Next, two specific primers were used to amplify the ligation product. PCR products were then denatured by heat and the single strand DNA was cyclized by splint oligo and DNA ligase. After that, 50 bp single‐end sequencing was performed on the prepared library by BGISEQ‐500. At least 20 M clean reads of sequencing depth were obtained for each sample.

#### 4.3.2 RNA and GSEA analysis

RNA‐seq raw data were filtered to obtain clean data after quality control, including removing adaptors, reads with more than 10% unknown bases and low‐quality reads and aligned to the mouse genome (mm10) by HISAT239. Raw counts for each gene were calculated by Htseq40. StringTie was used to estimate the expression level of detected genes 41. To ensure a robust analysis, genes detected in less than half the samples of each group were not taken into consideration for the DEG calculation. EdgeR was used to evaluate the statistical significance of DEGs with raw counts, and the additive linear model was used to compensate the batch effect42. DEGs were defined as genes with FDR < 0.01 and log2 fold change larger than 1 (upregulation) or smaller than −1 (downregulation). For GSEA analysis, we accessed the NCBI GEO repository to obtain a list of upregulated and downregulated genes for both CD21^−^CD23^−^ ABC (Russell Knode et al., 2017; GEO number: GSE81650) and CD11c^+^CD11b^+^ABC (Rubtsov et al., 2011; GEO number: GSE28887) and constructed a ABC signature gene list. GSEA analysis was performed using GSEA 4.1.0 software (Mootha et al., 2003; Subramanian et al., 2005).

### 4.4 Flow cytometry

Cells from spleen, lung, and BM were harvested and passed through a 70uM nylon mesh, washed, and stained in FACS buffer [0.5% Bovine Serum Albumin, 0.01% sodium azide (Sigma‐Aldrich) in PBS]. Cells were stained with amine reactive viability dyes to exclude dead cells (Invitrogen) and were blocked with anti‐FcR (2.4G2) and NMS (normal mouse serum). Surface antigens were stained with fluorochrome conjugated antibodies. Antibodies used were as follows: Alexa 700 anti‐CD19 (6D5), FITC B220, PerCp Cy5.5 CD23, APC‐efluor 780 CD21/35, IgD(11‐26c), BV650 IgM, PE CD95 (Fas, Jo2), APC GL7, and BV421 CD138. Following surface staining, cells were fixed and permeabilized with BD Cytofix/Cytoperm (BD Bioscience) kit following manufacturer's protocol for intracellular staining of FITC IgA (Southern Biotech), IgG2b, IgG2a/c, and PE‐Cy7 IgG1 isotypes (Biolegend). Antibodies were obtained from eBioscience, Biolegend, or BD Bioscience. Stained cells were acquired on an LSRII flow cytometer (BD) and analyzed using FlowJo analysis software. For T‐bet staining, cells were fixed and permeabilized using the FoxP3 fix/perm kit (eBioscience) following manufacturer's protocol and stained with BV421T‐bet (Biolegend). Antibodies were obtained from eBioscience, Biolegend, or BD Bioscience. Expression levels of different markers analyzed by flow cytometry are shown as MFI (Median Fluorescence Intensity) or nMFI (normalized MFI). To correct for batch effects while pooling data from different experiments, we normalized MFI by dividing each data point within an experiment by the average MFI of non‐Responding B cells. nMFI = MFI/(average MFI of non‐Responding B cells).

### 4.5 ELISA

MaxiSORP plates (Nunc) were coated overnight at 4 degrees with influenza PR8 in carbonate buffer, washed with PBS containing 0.05% Tween and blocked with PBS containing 1% BSA and 0.01% Tween for 1 hour at 37 degrees. Serum samples were serially diluted in PBS containing 1% BSA and 0.01% Tween and incubated for 2 h at 37°C. After washing, HRP‐conjugated Abs specific for mouse IgM and total IgG (Southern Biotechnology Associates) were added to plates and incubated 1 h at 37°C. After washing, TMB buffer (Thermo) was added, and color development was stopped with 2 M sulfuric acid solution. The OD reading of the color reaction was measured at 492 nm. EC_50_ IgM and total IgG titers were defined by half of the OD reading of the lowest dilution.

### 4.6 MDCK culture

MDCK (source) cells were seeded at 0.25 × 10^6^ cells (Gross et al., 2017) in a T‐75 flasks and incubated 37 °C in 5% CO_2_ for 2 days or until they reach 80%–90% confluency. Cells were then washed with PBS, incubated with Trypsin at 37°C for 10 mins and FBS was added to inactivate trypsin. Cells were washed and seeded at 0.25 × 10^6^ cells in T‐175 flasks. MDCK cells were allowed to reach 80%–90% confluency and seeded at 0.7 × 10^6^/well in 6‐well plates for plaque neutralization assay.

### 4.7 Plaque neutralization assay

6‐well plates were seeded with 0.7 × 10^6^ MDCK cells and were allowed to reach 120% confluency (2–3 days of culture). Heat inactivated serum was serially diluted and PR8 was diluted at 150 PFU/ml in 1% Bovine Albumin Fraction V (BSA), 100 U/ml penicillin, 100 μg/ml streptomycin, 100 μg/ml CaCl/MgCl_2_ solution in PBS. Viral and serum dilutions were mixed and incubated at 37°C for an 1 h (Gross et al., 2017). 6‐well plates were washed, inoculated with virus‐serum mixture, and incubated at 37°C for 2 h. Plates were rocked every 15 minutes to prevent drying of MDCK cells. Two milliliter of agar overlay medium composed of 0.6% agarose, 0.01% DEAE Dextran, 0.099% NaHCO₃, 1 μg/ml TPCK Trypsin, 50% MEM, and 18% H_2_O solution were added to each well and the plate was incubated at 37°C for 48 h. After plaques are formed, plaques were fixed with a 4% formaldehyde solution and incubated at room temperature for 1 h. Plaques were stained with a crystal violet solution for 15 mins at room temperature and plaques were counted.

### 4.8 Statistics

Unpaired, two‐tailed, Student's *t*‐test was used to assess statistical significance between the means of two groups, with *p* < 0.05 considered significant. Analysis was done using Prism (Graphpad) software. Error bars in the figures represent the standard error of the mean. Significance in the figures are indicated as *p < 0.05, **p < 0.01 and ***p < 0.001.

## AUTHOR CONTRIBUTIONS

O.K.U., P.D., and S.L.S. wrote the manuscript with assistance from A.M.R. and S.L.T. S.L.S and O.K.U conceived the project. O.K.U. and P.D. designed and analyzed the experiments. O.K.U carried out experiments with help from P.D., W.Z., Y.K., J.L., C.H.C., and S.L.T. All authors contributed to editing, read and approved the submitted version.

## CONFLICT OF INTERESTS

The authors declare that they have no competing interests.

## Supporting information


Figure S1

Figure S2

Figure S3

Figure S4

Figure S5

## Data Availability

Data available in article as supporting information.
