# Supplementary figures and images for: IgD + age‐associated B cells are the progenitors of the main T‐independent B cell response to infection that generates protective Ab and can be induced by an inactivated vaccine in the aged
Source: Aging Cell. 2022 Sep 2;21(10):e13705. doi: 10.1111/acel.13705 (PMC9577953; doi:10.1111/acel.13705)

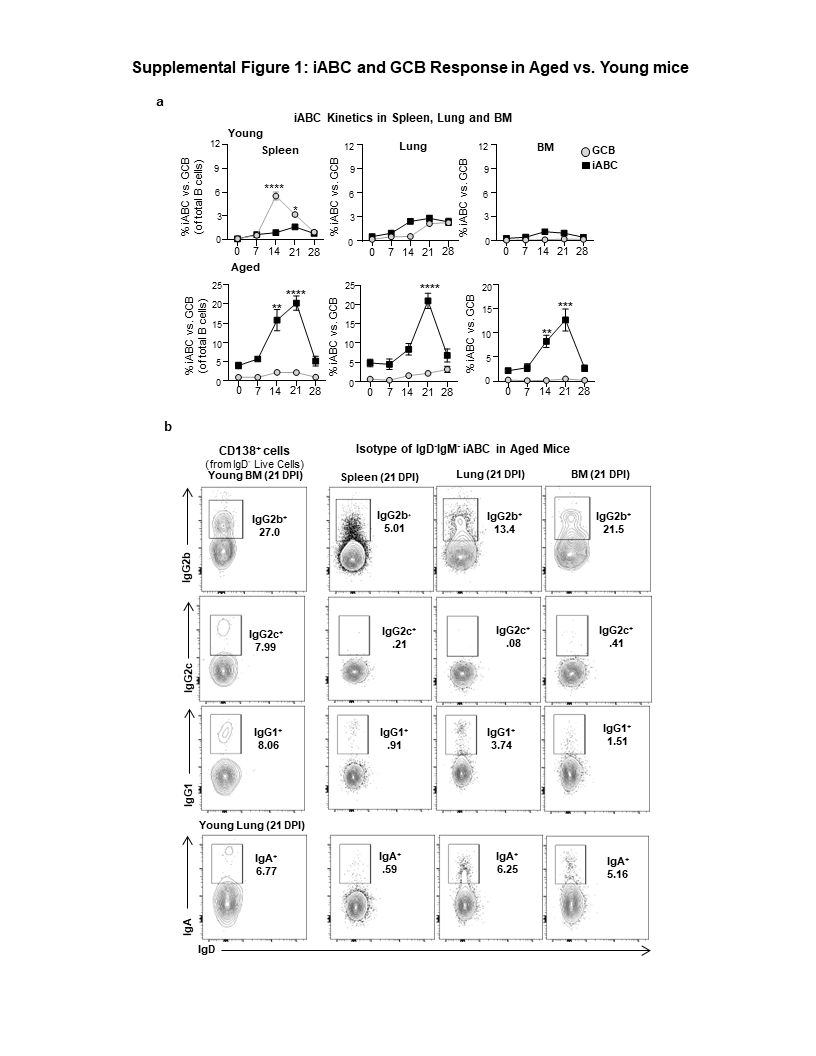

Supplement: Supplementary file 1 — Figure S1 [file ACEL-21-e13705-s003.png]

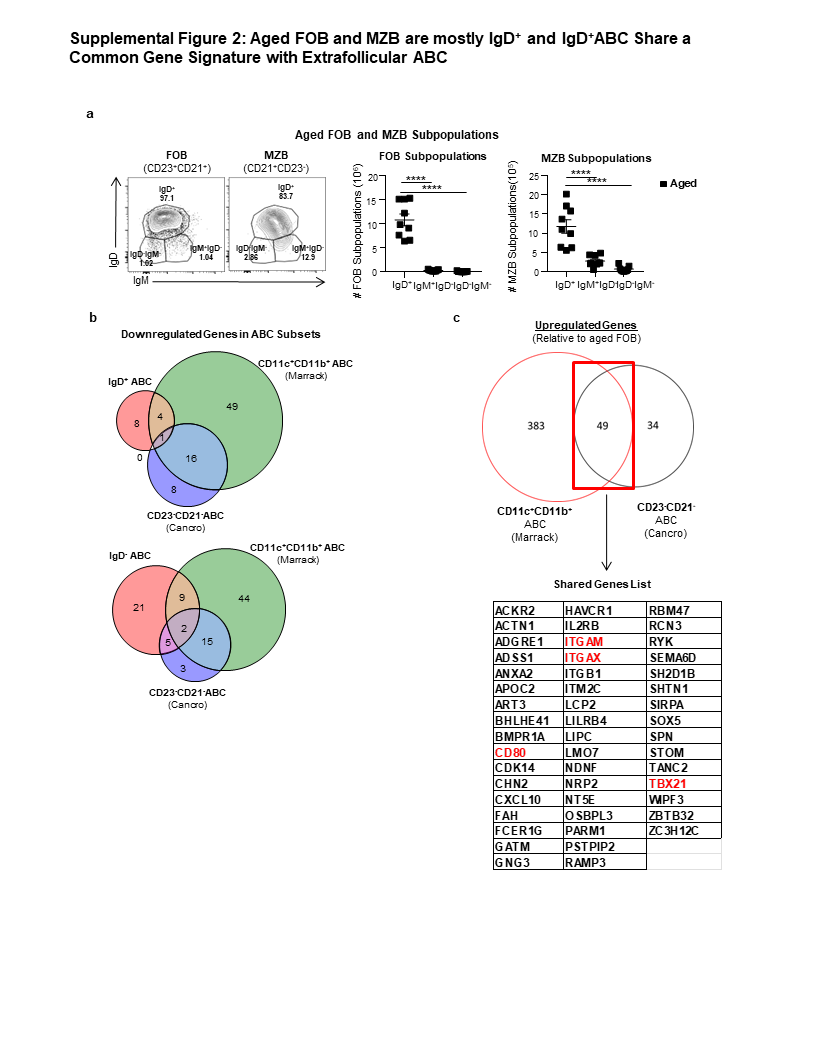

Supplement: Supplementary file 2 — Figure S2 [file ACEL-21-e13705-s001.png]

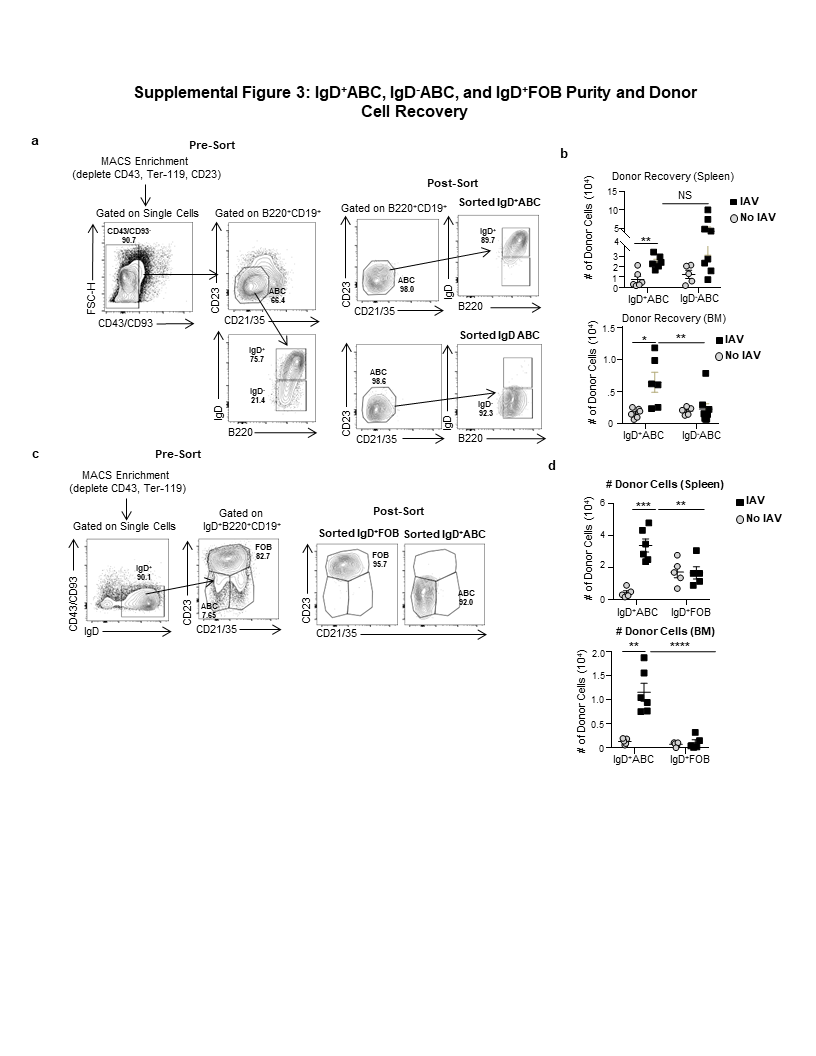

Supplement: Supplementary file 3 — Figure S3 [file ACEL-21-e13705-s005.png]

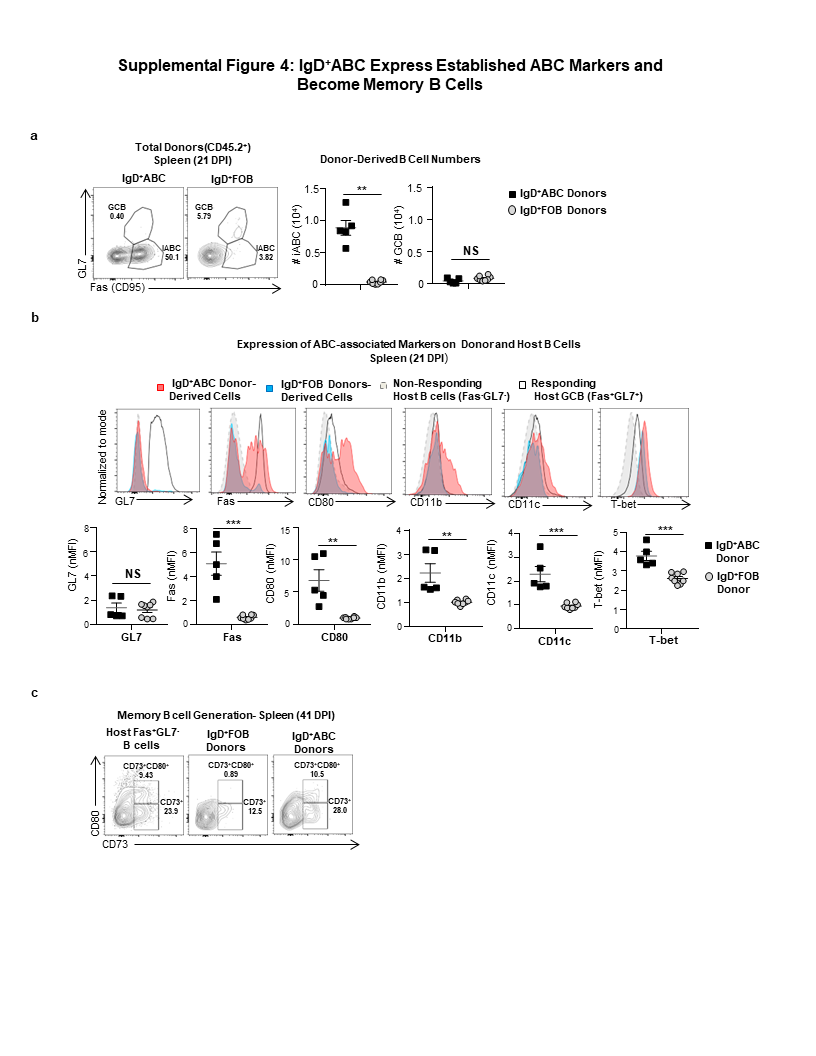

Supplement: Supplementary file 4 — Figure S4 [file ACEL-21-e13705-s004.png]

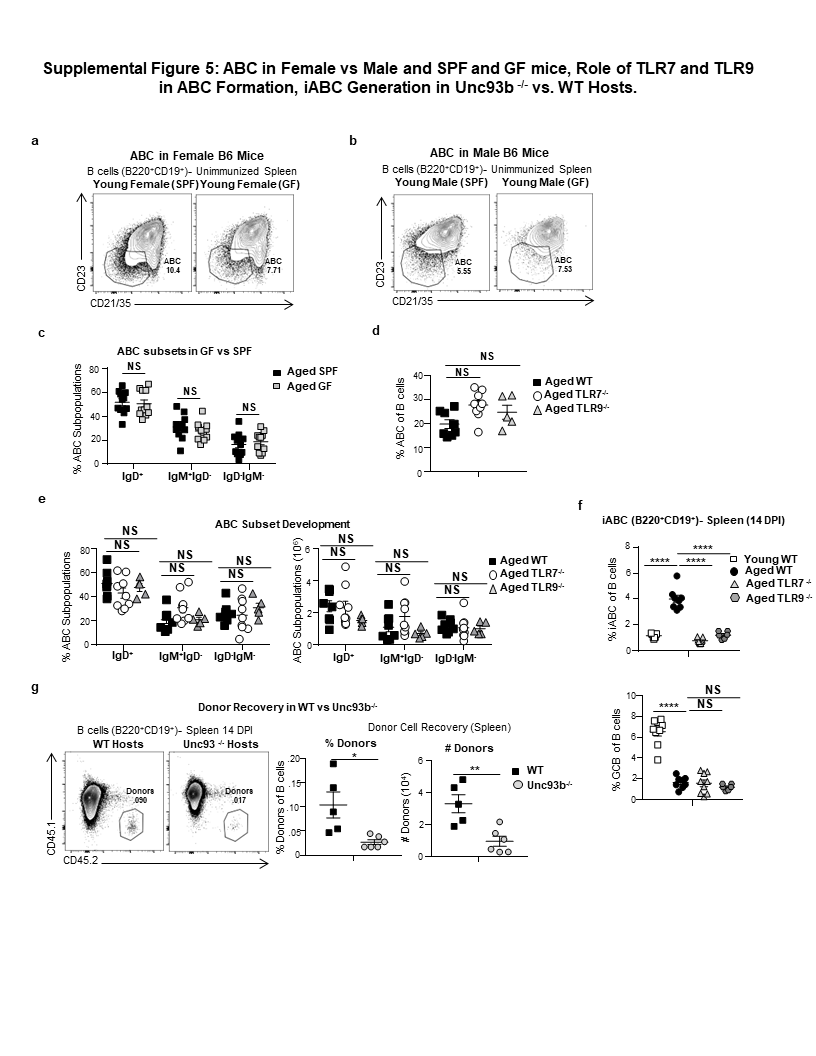

Supplement: Supplementary file 5 — Figure S5 [file ACEL-21-e13705-s002.png]
